# Organising Nurse Work Environments: (Reshaped) Roles of Nursing Teams—A Qualitative Descriptive Study

**DOI:** 10.1111/jan.70093

**Published:** 2025-07-28

**Authors:** Janet Bloemhof, Jeannette Knol, Marjon Van Rijn, Bianca M. Buurman, Mirko Noordegraaf

**Affiliations:** ^1^ Department of Nursing Staff Tergooi Medical Centre Hilversum the Netherlands; ^2^ Utrecht School of Governance Utrecht University Utrecht the Netherlands; ^3^ Department of Internal Medicine, Section of Geriatric Medicine Amsterdam UMC Amsterdam the Netherlands; ^4^ Department of Medicine for Older People Amsterdam UMC Amsterdam the Netherlands; ^5^ Amsterdam Public Health, Aging & Later Life Amsterdam the Netherlands; ^6^ Amsterdam University of Applied Sciences, Research Group Integrated Complex Care, Faculty of Health Center of Expertise Urban Vitality Amsterdam the Netherlands

**Keywords:** capabilities, nurse work environment, nurses, nursing teams, organising professionalism, professional development, professionals

## Abstract

**Aim:**

To explore how nursing teams (co)organise their work environment by going beyond caregiving.

**Design:**

A descriptive qualitative study with a phenomenological approach.

**Methods:**

Semi‐structured group interviews were conducted in 2022 with nurses and managers from 18 nursing teams in a general hospital located in the Netherlands. In each group interview, 2–3 participants per team took part. The interviews were audio‐recorded, transcribed verbatim, and analysed using thematic analysis.

**Results:**

The analysis identified four main themes contributing to a more supportive work environment: (1) embracing diversity, (2) stretching nursing roles, (3) raising voices, and (4) aligning nurses and managers. These themes show how nursing teams go beyond caregiving and actively shape and co–organise their work environment.

**Conclusion:**

Teams that extend their roles create more supportive work environments, enhancing patient care and professional development. These findings contribute to the understanding of organising professionalism in nursing and provide insights for nursing teams striving to improve their work environments.

**Implications for the Profession:**

Nursing teams can use the four themes—as team features—to reflect upon their organising roles and engagement with their work environment. Our findings offer practical insights for nurses with responsibilities in areas such as team development and leadership. They can focus on team diversity, voicing, stretching roles, and organisational alignment, and facilitate their teams to become more assertive.

**Reporting Method:**

The Consolidated criteria for Reporting Qualitative research guideline was followed.

**Patient or Public Contribution:**

No patient or public involvement.

**Summary**

- What already is known
  ○ The nurses' work environments affect both the quality of care and nurses' job satisfaction, and need strengthening due to increasing care complexity and workforce shortages.
  ○ Research on improving the nurses' work environments has mainly focused on nurse leaders and organisations, with limited attention to the role of nursing teams themselves in shaping their work environment.
  ○ Nurses can contribute to their work environment as ‘organising professionals’ by collaborating, leading, coordinating, and taking responsibility—going beyond direct caregiving tasks.
- What this paper adds
  ○ We identified four key team‐level features—embracing diversity, stretching nursing roles, raising voices, and aligning nurses and managers—that enable nursing teams to shape a supportive work environment.
  ○ This study demonstrates that reshaping the nursing work environment is an active, intentional process involving collaboration within teams and between nurses and managers.
  ○ This study showed how the interplay between these team features—such as diverse perspectives and expanded professional roles—reinforces nurses' ability to co‐organise their work environment.
- Implications for practise and policy
  ○ The four team features can help nursing teams reflect on their organising practices and identify areas for development, such as team diversity, voicing, role stretching, and alignment.
  ○ For management, it is important to recognise, appreciate, and enlarge nursing roles that extend beyond direct caregiving to improve caregiving.
  ○ Future research could further define the organising capabilities linked to the identified themes and explore how nurses can be supported in developing these skills.

## 1 Introduction

Nurses' work environments are essential for nursing practice (Beiboer et al. 2023; Lake et al. 2020; Wei et al. 2018). They encompass organisational characteristics of work settings that either facilitate or constrain professional nursing practice (Lake 2002). Increasing complexity of care caused by higher patient acuity, the advancement of new technology, and an ageing patient population (Haddad et al. 2023; Heikkilä et al. 2021; Van den Heede et al. 2019), coupled with labour market tightness, places increasing demands on nurses' work environments (International Council of Nurses 2021; World Health Organization 2020). Strengthening nurses' work environments is essential for coping with these demands and might enhance job satisfaction for nurses and improve the quality of patient care (Aiken et al. 2023; Kohnen et al. 2023).

While most research on improving nurses' work environments is approached from the perspective of nurse leaders and organisations, there is a lack of research investigating nurses' contributions to enhancing their work environments (de Kok et al. 2023; Maassen et al. 2021; Wei et al. 2018). As frontline healthcare providers, nurses are uniquely positioned to cultivate and maintain their work environments. This necessitates a more comprehensive role for nurses that focuses more on the organisation of care and goes beyond caregiving. In the literature, this development is referred to as ‘organising professionalism’ (Noordegraaf 2015). Organising is then seen as a natural part of professional action. Organising professionals can (co)organise their work by collaborating, leading, coordinating, and being accountable for their actions (Noordegraaf 2015). Such organising is essential for enabling nurses to address the increasing demands on their work environments more directly and hands‐on. By actively shaping care processes, nurses can foster collaboration and efficiency, reduce workload pressures, and enhance job satisfaction and patient outcomes.

Our research focuses on the nurses work environment to overcome the emphasis on either managers/leaders and healthcare professionals. Anticipating and actively shaping one's work environment can be a part of day‐to‐day professional action. This study builds on a previous study investigating the impact of implementing a professional practice model on the nurses' work environment (Bloemhof et al. 2021). Considering that nursing work occurs within a team setting, and effective teamwork is acknowledged as a fundamental factor in ensuring patient safety (Alanazi et al. 2021; Bragadóttir et al. 2023; Jomaa et al. 2021; Rahn 2016), we direct our attention to nursing teams rather than individual nurses. In doing so, this study aims to deepen our understanding of how nursing teams (co)organise their work environments by going beyond caregiving.

## 2 Background

Over the past decades, the field of nursing has undergone significant changes. At the start of the 21st century, nursing was considered a semi‐profession (Freidson 2001), with a strong focus on caregiving tasks. Since then, the role of nurses has evolved. The current nursing curriculum notably emphasises problem‐solving skills, evidence‐based practice, interprofessional collaboration, prevention, and leadership (LOOV 2023). Nowadays, nursing is recognised as a more fully professional discipline (Ruijters and Simons 2020), encompassing responsibility for nurses' work environments.

Despite being recognised as more full‐fledged professionals, in practice, the emphasis is often on the direct patient‐related dimensions of patient care rather than the indirect aspects, such as quality improvement and education and training (Allen 2018). This is also how others often perceive nurses. During the recent COVID‐19 crisis, it turned out that nurses were predominantly portrayed as heroes at the bedside (Boulton et al. 2022). On the other hand, there is unprecedented attention to nurses as professionals, recognising their capabilities beyond traditional caregiving, for example, the differentiated clinical‐scientist roles (Martini et al. 2023), their work as public opinion leaders (van Wijk et al. 2022) and in nurse councils (Verhoeven et al. 2022).

In the literature, this changing role (beyond traditional caregiving) is described as organising professionalism (Noordegraaf 2011; Noordegraaf 2015; Noordegraaf 2020). Organising professionals are characterised by the following features: (1) they actively participate in collaboration, crossing boundaries between departments, for example, by linking professional standards to organisational standards and policy frameworks; (2) they take responsibility and autonomously shape their work, for example, by anticipating dilemmas and challenges instead of passively letting them evolve; (3) they embrace proactive coping strategies, for example, by using ‘voice’ instead of a reactive approach of continuously working more intensively, without pause or reflection; and 4) they integrate substantive expertise (e.g., correct protocols) with enhanced organisational skills (e.g., prioritising tasks, managing workflows, exercising leadership).

In this way, they transcend the role of bedside caregivers and adopt more comprehensive roles as part of their nursing profession. Adopting these roles also helps face organisational, policy, and social complexities that nurses and healthcare organisations encounter. These complexities are not merely organisational or managerial; they are (also) professional and part of nursing work.

Addressing these complexities and realising nurses' potential as organising professionals requires a shift towards organising professionalism supported by healthcare organisations. This involves changes in work practices, with managers and executives establishing clear frameworks—such as protocols, decision‐making structures, and resources—and facilitating organisational change processes through targeted support, training, and open communication (25). Many kinds of programmes have been established throughout the Western world to encourage and support healthcare organisations in strengthening professional practice environments for nurses. One of the most widely recognised programmes is the Magnet Recognition Programme (American Nurses Credentialing Center 2021), comprising five key components: (1) transformational leadership, (2) structural empowerment, (3) exemplary professional practice, (4) new knowledge, innovations, and improvements, and (5) empirical outcomes (American Nurses Credentialing Center 2021). These components illustrate that improving the nurses work environment requires a collaborative effort throughout the entire organisation. The so‐called ‘Excellent Care’ programme in the Netherlands has been developed based on the principles established by Magnet hospitals (Verpleegkundigen en Verzorgenden Nederland 2020).

Within the Excellent Care programme, nurses' perceptions of their work environment are important. Numerous definitions of a supportive work environment exist, each encompassing distinct dimensions (Maassen et al. 2021). In our research, we operationalise the nursing work environment by employing the eight magnet characteristics derived from the Excellent Care model. These characteristics include (1) working with clinically competent peers, (2) collaborative nurse–physician relationships, (3) clinical autonomy, (4) nurse manager support, (5) control over nursing practice, (6) perceived adequacy of staffing, (7) support for education, and (8) culture in which attention for the patient is paramount.

The nurses work environment is important for nursing practice, as it influences the quality of care and job satisfaction. Research also shows that effective teamwork is linked to improved patient outcomes and job satisfaction (Alanazi et al. 2021; Bragadóttir et al. 2023; Jomaa et al. 2021; Rahn 2016). However, despite their interconnected nature, nurses work environment and teamwork are often studied independently.

## 3 The Study

With our research, we aim to contribute to the understanding of how nursing teams can perform roles beyond caregiving, particularly by actively jointly participating in improving their own work environment. We address the following research question: How do nursing teams (co)organise their work environment by going beyond caregiving?

## 4 Methods

### 4.1 Design

In this descriptive qualitative and explorative study, we adopted a phenomenological approach (Polit and Beck 2021) to gain an in‐depth understanding of how nursing teams experience and (co)organise their work environment. The Magnet characteristics provided a conceptual foundation for exploring supportive work environments in nursing. To collect data, we conducted semi‐structured group interviews (DiCicco‐Bloom and Crabtree 2006; Kallio et al. 2016). These group interviews enabled nursing teams to narrate their perspectives on their work environment collectively. The interactive nature of group discussions facilitated dialogue and reflection among participants, enabling nursing teams to explore and articulate their experiences and insights in greater depth (Polit and Beck 2021).

### 4.2 Study Setting and Recruitment

The study was conducted in 2022 in a Dutch general hospital with 370 beds and 2600 employees, including 750 nurses. All nursing teams (*N* = 20) were invited to participate in an interview with two nurses and the team manager. We used purposive sampling as the nurses selected for participation were the ‘Excellent Care champions’, appointed to each department during the Excellent Care program (Bloemhof et al. 2021). If these champions were unavailable or no longer employed, we asked the team to suggest other nursing colleagues. We selected the Excellent Care champions because we saw these group interviews as an initial step towards enhancing the nurses' work environment within the teams. We invited the managers because we believe, and research has shown, that they play a crucial role in creating a positive nurses' work environment. The champions and their manager were approached via email to schedule an appointment for the group interview. Separate group interviews were conducted for each team. The interviews were held in person at a hospital location and were audio‐recorded with the participants' permission. JB and JK facilitated the group interviews.

### 4.3 Data Collection

Referring to the literature (Maassen et al. 2021; de Brouwer et al. 2014; Kramer and Schmalenberg 2004), we developed a topic list (see appendix 1) incorporating the ‘Essentials of Magnetism’. This framework was utilised to stimulate reflective discussions, aiming to uncover narratives about how nurses perceive their work environment. The interviews were conducted by the first and second authors (JB and JK). Initially, JB conducted two interviews. This was followed by an interview conducted by JK, with JB observing. No changes were made to the topic list based on these initial interviews, but emphasis was placed on improving follow‐through questioning techniques. Subsequently, JB and JK alternated conducting the interviews, with JB conducting the majority. Group interviews were conducted with 18 out of 20 teams. Despite multiple attempts, appointments could not be arranged with the remaining two teams. The 18 interviews lasted between 31 and 60 min each. The number of participating nurses and managers varied, as given in Table 1. All group interviews were held in Dutch, audiotaped, and transcribed verbatim.

**TABLE 1:** Characteristics of the participants.

Team	Participant 1	Participant 2	Participant 3	Total
1	Manager	BN^a^	VN^b^	3
2	Manager	BN	VN	3
3	Manager	BN	Masternurse	3
4	BN	VN		2
5	Manager	BN in training	VN	3
6	Manager	BN		2
7	Manager	VN		2
8	Manager	BN	VN	3
9	Manager	VN	VN	3
10	Manager	BN		2
11	Manager	BN		2
12	Manager	BN	VN	3
13	Manager	Management trainee	BN	3
14	BN	BN		2
15	Manager	BN		2
16	Manager	Management trainee	VN	3
17	Manager	VN		2
18	Manager	VN		2
			Total	45

^a^ Bachelor of Science‐trained nurses.

^b^ Licensed vocational‐trained nurses.

### 4.4 Data Analysis

A team of five researchers conducted a thematic analysis, guided by Braun and Clarke (2021, n.d.): JB (junior nurse researcher), JK (senior nurse researcher), MvR (senior nurse researcher), BB (professor in nursing science), and MN (professor in management and organisation science). Although the eight Magnet characteristics were used to structure the interview guide, we applied a hybrid (abductive) approach to the thematic analysis: the analysis began inductively with open coding to allow for unexpected insights, while the overarching structure was informed by existing concepts. Themes were developed from the data at a semantic level. First, interviews were transcribed verbatim (JB). Transcripts were repeatedly read and discussed, developing initial coding ideas (JB, JK, and MvR). Second, JB and MvR double‐coded two interviews to identify initial codes. They assessed similarities and discrepancies in codes. Third, JB sorted initial codes into potential themes and collected relevant coded segments. Using iterative constant comparison, JB integrated new data, structured relevant data for each theme, and crafted preliminary descriptions. Fourth, themes were reviewed alongside the codes and transcripts to accurately reflect the dataset (JB, JK, and MvR). Fifth, themes were refined, named, defined (JB), and reviewed with the research team (JB, JK, MvR, BB and MN). Following additional team discussions, the themes underwent further revision and finalisation. The authors met regularly during the analysis to discuss emerging ideas and theoretical insights. Finally, JB, JK, and MvR drafted a report including vivid quotes to illustrate the themes, synthesising the analysis into a coherent narrative. MAXQDA 2022 (VERBI Software 2021) was used to support all stages of coding and analysis.

### 4.5 Rigour and Reflexivity

The concept of *organised professionalism* served as a theoretical foundation that informed the study's background and helped contextualise the findings. The research process was highly systematic and transparent, ensuring methodological rigour. Semi‐structured group interviews were carefully designed to capture both nursing and managerial perspectives, and data analysis followed an iterative process using thematic analysis. To enrich interpretation, theoretical triangulation was applied in the later stages by relating the findings to the concept of *organised professionalism*.

The data analysis process was highly collaborative, with continuous discussions within the research team ensuring a comprehensive and multi‐perspective approach. This engagement facilitated a thorough exploration of diverse viewpoints, strengthening the depth of data interpretation. Throughout the process, the principal investigator took detailed memos to document reflections and potential biases.

### 4.6 Ethical Considerations

In line with Dutch law (CCMO), no approval of an ethics committee was applicable as this study included staff and because no medical or patient data were collected, and individuals were not subjected to invasive or demanding regimes (CCMO 2024). The board of directors of the hospital approved the conduct of the study. Participants gave consent before the audio recording of the group interviews. Data were stored according to Dutch Data Protection Laws (Autoriteit Persoonsgegevens n.d.) | autoriteit persoonsgegevens.). The study adhered to the Declaration of Helsinki (World Medical Association 2024).

## 5 Findings

Our analysis revealed both similarities and differences within and across teams in how they engage with their work environment and how they reshape it. From these varied experiences, we identified four key themes that are crucial for teams to influence and reshape their work environments to advance nursing professionalism: (1) embracing diversity, (2) stretching nursing roles, (3) raising voices, and (4) aligning nurses and managers. We discuss each theme individually, highlighting the experiences of the teams we studied.

### 5.1 Embracing Diversity

Participants frequently highlighted team diversity in the group interviews, particularly regarding age, work experience, and educational background. One nursing team's approach to assigning specific focus areas to nurses exemplified this diversity. During this process, it became evident that an older colleague was experiencing difficulties with the focus area assigned to him, which was the nursing process. Rather than reallocating tasks, the team members collectively chose to support their colleague in overcoming these challenges. They simultaneously leveraged his specific expertise in areas such as intravenous cannulation and mentoring nursing students. This approach allowed the colleague to grow in his assigned role and highlighted the team's ability to embrace diversity effectively. This example illustrates how teams can harness diversity to positively influence their work environment, a theme that emerged in various forms throughout our analysis.

The diversity in age and experience within nursing teams led to both challenges and opportunities. Some teams highlighted the ‘unique contributions’ of each team member, while others often framed discussions in terms of an ‘us versus them’ attitude. Whether and how teams are able to deal with diversity seems to matter.

In nursing teams, common terms like ‘youngsters and oldies’ or ‘the young and old guard’ are frequently used to denote age and work experience differences. Some teams openly express concerns that younger nurses must gain the necessary proficiency in handling patients' illnesses or anticipating needs. As stated by one nurse:

They [the younger nurses] may not always know exactly how to handle things when it comes to patients' illnesses or are unable to anticipate properly. (Nurse Team 12)

The critique of younger nurses' work practices was further elaborated:

And then you hear from a patient's relative that her husband hasn't been washed for two days because they [the younger nurses] give him towels and do nothing else. Sometimes I think I want to have experienced professionals; then I'm almost sure it will be done properly. (Nurse Team 16)

In several teams, younger nurses perceived older colleagues as somewhat ‘inflexible’ or ‘resistant to change’, suggesting that newer team members were often more open to adopting new ways of working.

As opposed to polarising generations, in some teams, nurses actively reflect on their generations' unique characteristics. This reflection highlights their willingness to understand generational differences and shows their commitment to improving collaboration within the team.

Not because they [the younger nurses] are better, but because they are not as preformed yet. They do not carry the baggage we, as older nurses, have. They are very initiative‐driven and still have all that energy, while we are doing it the same way for years. (Nurse Team 2)

In another team, team members mention that they have a very young team, but they are all eager to learn and maintain high standards:

We have many new colleagues, but everyone is well‐trained, and we have made good agreements with each other. Everyone takes on their working groups, and everyone truly strives for quality. Yes, I really think things are going well. (Nurse Team 5)

While age and experience diversity were common topics, another aspect of diversity emerged with the introduction of role distinction based on educational background.

In 2018, the hospital introduced role distinction between licensed vocational‐trained nurses (VNs) and Bachelor of Science‐trained nurses (BNs). Although the differentiation aimed primarily at enhancing quality improvement, research, and work organisation rather than the complexity of patient care, some nurses experienced discomfort and a sense of rejection. One nurse mentioned:

I do receive feedback that VNs are quite unhappy with how much attention, in their perception, is given only to BNs. When there's a call for interesting projects, they always ask BNs, as if they [VNs] have nothing to contribute. (Nurse Team 6)

Conversely, other teams have positively integrated the professional practice of BNs, allowing them to utilise their education in practice. For instance, before role distinction was introduced, one team (Team 16) worked with senior nurses who could be vocational‐trained or Bachelor of Science‐trained. The former senior nurse of that ward mentioned that many tasks fell solely on her shoulders. She felt relief after role differentiation was implemented, no longer having to ‘carry the load alone’. Now, in this team, VNs and BNs collaborate on a project to improve the discharge procedure, complementing each other well. BNs search for literature and develop ideas using online project management tools, while VNs contribute their patient care expertise and leverage the connections they’ have built over the years.

Experiences with BNs within nursing teams vary. One nurse noted a decline in team communication and trust since the introduction of role distinction, stating:

The starting point was that we were pretty good at communicating as a team, and over the past year or so, it has become inferior. Nurses talk about each other instead of with each other; not everyone feels trusted. The BNs has taken a hit. (Nurse Team 1)

In contrast, another nurse observed improvement and growth within the team, remarking:

I think we can still grow better as a team, but I do notice that we are on the rise due to the addition of the BNs. (Nurse Team 16)

As demonstrated in our opening example and throughout the analysis, teams that embrace diversity are able to recognise and utilise individuals' strengths, competencies, and personal characteristics. Differences and similarities are consciously acknowledged and valued, utilised in an informal, natural way in daily work. Embracing diversity appears to serve as a common ground for teams to ‘reshape’ their work environment.

### 5.2 Stretching Nursing Roles

One nursing team in our study demonstrated how proactive efforts can reshape the work environment and enhance patient care. Observing an increasing number of older patients across hospital units, they took the initiative to address the unique challenges faced by this vulnerable group. Motivated by research on geriatric care, the team developed a specialised educational programme tailored to improving the care of older patients. The team extended their expertise hospital‐wide rather than limiting their efforts to their department. They organised training sessions to share their knowledge with other teams. This initiative improved the quality of care for older patients and highlighted how nursing teams can expand their roles beyond traditional caregiving tasks and beyond their own ward.

This example sets the stage for exploring how nursing teams balance their primary focus on patient care with broader responsibilities, such as improving care quality and engaging in professional development. It illustrates the potential for nurses to stretch their roles and positively influence their work environment, a theme that emerged consistently across our data.

All nursing teams indicate that their primary focus is on patient care:

The patient is our priority. Basic care and patient care always come first, no matter how busy we are. It always continues, even if it means we don't have a coffee break and only have lunch very late. (Nurse Team 13)

Finding time for tasks not directly related to patient care remains challenging for nurses.

One nurse described it as a ‘blur’ encompassing everything outside the department:

I know there is a nursing platform; I know there are protocols. I get an email, and another email, and again an email. I read something on the intranet; I know a lot happens outside the ward, but I don't have time to sit quietly and read it carefully. You miss a lot. And sometimes, I start to open my email and read, and then I'm halfway through the text, and my beeper goes off. And I never move on. (Nurse Team 4)

In addition to finding time for tasks not directly related to patient care, nurses' proactivity and sense of ownership are also frequently mentioned as important factors:

Everything remains in the air because no one feels responsible for anything, and everyone is too tired to follow through on tasks. Meanwhile, everything remains unresolved. (Nurse Team 8)

Within the teams, there is discussion regarding whether the entire team should engage in tasks not directly related to patient care, such as efforts to improve the quality of care. Typically, a few nurses assume these responsibilities, highlighting the need for more ‘proactive’ members within the team. Furthermore, it is crucial that those who are less proactive refrain from criticising their more proactive colleagues.

Despite the challenge of finding time, there is a general willingness among teams to consider ways to improve patient care:

We never discuss how we can make care more patient‐centered. While it may already be good, it's still interesting to see how we can improve it. (Nurse Team 14)

And team members want to grow as a team:

As a team, we should think about how we are currently performing and how we can grow together. I want everyone to contribute, show leadership, and look ahead together. (Nurse Team 3)

For sure, nursing teams identify many processes that can be improved to enhance patient‐centredness, such as improving discharge procedures and addressing the continuation of treatment for very ill patients. One nurse stated:

… and then I think it has to stop at some point. And then the patients say to us: it's not necessary anymore, it's okay. And when we relay that to a doctor, it's: but we can still do this and that. Then, they persuade the patient so convincingly that they give in again. In terms of nursing care, I think the care culture is excellent towards the patient, but there's too much medical treatment and insufficient consideration for the patient's wishes. (Nurse Team 19)

However, it remains challenging for nurses to tackle this issue. This team mentioned that nurses discuss these kinds of cases and have ‘substantial input’ with the doctors, but not when it comes to matters involving the (dis)continuation of treatment.

All teams acknowledge the abundance of educational opportunities available in the hospital, but everyone also agrees that these opportunities are not utilised to their full potential. Often, only a few team members take advantage of these educational opportunities. The training opportunities and budget allocation are also often not discussed within a team. One nurse noted:

Attending a conference or a workshop enhances your professionalism and allows you to gain more knowledge. I find it very valuable, and I'm curious why others don't see it as valuable. (Nurse Team 13)

However, she also indicated that she did not raise this question within the team to initiate a discussion.

In contrast, another team (Team 5) took a more proactive approach, similar to our opening example. One of the nurses requested to manage the education budget. She and a colleague oversee the budget and its allocation, monitor available educational opportunities such as workshops and conferences, and assess the team's needs and interests. As a result, the budget is now fully utilised and spent, unlike in previous years.

This approach contrasts with another team's (Team 4) proceeding. When asked about their efforts to keep up with developments in the nursing profession and whether they read professional journals or discuss educational opportunities:

Rarely, very rarely. You do your thing, go home, and return to do your thing again. If you have questions or need something, ask the doctor or look it up. We don't collectively discuss or take the time to talk about it. Occasionally, we receive clinical lectures from doctors; that's what we rely on. (Nurse Team 4)

Notably, both teams (4 and 5) have the same team manager, highlighting how team dynamics and individual team members' initative can influence the stretching of nursing roles.

What stands out is that teams that stretch nursing roles, like the one in our opening example, proactively create time and space to focus on more than just patient care, including collaboration with other disciplines. They take responsibility for addressing challenges in their work environment, looking beyond their department. This broader perspective allows them to apply their competencies in areas beyond patient care, helping to shape their work environment and contribute to improvements on a wider scale.

### 5.3 Raising Voices

One of the smaller departments faced staffing vulnerabilities, prompting management to propose a merger with another department. However, nurses perceived this planned merger as highly impactful for their team and raised various concerns, including ensuring the quality of patient care. To voice their concerns, the nurses wrote a letter to management and the Board of Directors, detailing potential risks and emphasising the need for a thorough merger evaluation. Their approach also involved engaging the nursing council to amplify their concerns. In response, a joint session was organised, during which the risks were discussed in‐depth, and concrete measures were agreed upon to address them. This example illustrates how voicing concerns can lead to meaningful dialogue and collaborative solutions, even in the face of significant challenges.

In the hospital, nurses have several opportunities to share their opinions and contribute to decision‐making processes through formal structures like the nursing council (strategic and tactical levels) and the nursing platform (operational level). Nursing teams acknowledge that these structures have improved over the years, providing more opportunities to voice their concerns.

When I compare it to a few years ago and now, I see that there have been many improvements as well. We now have a nursing council, and there's a nursing platform where you can raise issues. (Nurse Team 1)

Despite these improvements, many nurses still feel ‘disconnected’ from these initiatives. While they are generally aware that such initiatives exist and consider them to be of good quality, they often report having ‘little’ to ‘no personal knowledge’ of them nor ‘direct involvement’.

In many teams, only a few colleagues are involved in the nursing platform and council. Many are unaware of these structures and their differences. This lack of engagement often leads to negative perceptions, with decisions from the council and platform perceived as being made in an ‘ivory tower’. Consequently, these teams make minimal effort to engage with the platform or council to gain a clear understanding of their roles.

Conversely, some teams recognise the necessity of these structures to ensure their voices are heard. They view the nursing platform as a potential ‘networking hub’ representing all teams. For example, one team designated a nurse to ensure their presence on the platform. Another team divided the responsibility among several nurses, who took turns attending platform meetings.

Nursing teams have noted that issues are occasionally brought up for discussion during informal team meetings, often referred to as ‘coffee and locker room talk’. But once in the formal meeting, everyone tends to agree, despite having voiced complaints earlier.

Then, when you're sitting there with everyone, nobody says a word; it's all just agreement at that moment. Yet, not even half an hour before, they complained to us or anyone else in the department. In those moments, I think, is it because they don't feel a safe environment, are they afraid to speak up, or they are hesitant to express their concerns. (Nurse Team 3)

This nurse cites the ‘lack of a safe environment’ as a possible explanation for not speaking up, referring to not feeling at ease or sufficiently supported to voice concerns. Other reasons mentioned included ‘not recognizing opportunities’ to speak up, being ‘unaccustomed’ to forming and expressing opinions, and the challenge of ‘stepping out’ of one's ‘comfort zone’.

You've been doing the same job for many years, and suddenly, you must start thinking about it and forming an opinion. You need to have an opinion, show a bit of courage, and think, “How does that work”? (Nurse Team 18)

A concrete example from one team illustrates the importance of careful consideration in decision‐making. When a nursing department temporarily closed several beds, part of the team was forced to work on another ward. The team was given three options: (a) continue working entirely on their ward, (b) work partly on their ward and partly on another ward, or (c) move entirely to another ward. After submitting their preferences, the ward was closed entirely. The nurses asked for clarification and were told: ‘There's nothing we can do about it’. Despite claims that all options were considered, this led to disappointment and scepticism about the value of their input. One nurse said: ‘Why ask for our opinion if it doesn't matter anyway? They shouldn't have asked for our opinion at all’ (Team 5). This example shows that asking for input when there is no real choice can lead to a false sense of control over nursing practice. It underscores the need for careful attention to the process of control over nursing practice.

A perceived lack of efficacy further hinders the willingness of nurses to voice their opinions, as respondents doubted whether their input ‘would influence’ their organisation. For many nurses, having a say feels ‘abstract’ and ‘distant’, associated with formal structures like nursing councils rather than their daily practice. Discussing empowerment with nursing teams often reveals that many colleagues focus on direct patient care and avoid additional responsibilities. Even when paid opportunities to participate in decision‐making are offered, many nurses choose to go home after their shifts. As a result, having a say is perceived as an ‘add‐on’ rather than an integral part of bedside practice.

What stands out is that teams that bring their concerns to the right stakeholders and see their input considered, like the one in our opening example, present themselves as a cohesive collective rather than a group of individuals. They utilise existing ‘communication channels’, such as management and the Board of Directors, and other structures like the nursing council. By incorporating diverse voices and perspectives from within the team and beyond, they ensure their message is widely supported and reaches the appropriate audience. These teams pay attention to what is happening in their work environment and create opportunities to enact improvements. In doing so, they shift from only recognising issues to taking concrete steps that result in changes within their work environment.

### 5.4 Aligning Nurses and Managers

A team manager observed that their team scored low on development opportunities over an extended period. This specialised department offered limited options for professional growth within its specialisation, but opportunities existed for nurses to develop in complementary roles, such as becoming a lactation consultant. Recognising the need for improvement, the team engaged in discussions to explore ideas for development. Suggestions included creating educational videos and pursuing other initiatives to enhance their skills. The manager explained the options while clarifying that not all ideas could be implemented for every team member. Through these discussions, mutual understanding emerged. The manager gained insight into the professional domain of the nurses, while the nurses, in turn, developed an understanding of the managerial framework. This exchange led to conversations about development opportunities, culminating in a collaboratively developed training plan. The manager and the team worked towards a shared goal by working together within the defined parameters. The relationship between nurses and their managers plays a key role in shaping the work environment, as shown in the earlier example and extensive discussions on this topic during interviews with various teams.

It became clear that while managerial support is universally desired, the specific needs and expectations can vary significantly between different nursing teams and even among individual nurses. For some teams, the emphasis is on having an ‘approachable’ manager, where the sense of having an ‘open‐door policy’ is highly valued.

I know I can always walk in. I'm sure that if I'm really struggling with something and I drop by, she'll be there for me. Of course, it also depends on how you approach it yourself—maybe that's just how I am. But I always feel like I can turn to her, and that means a lot to me. (Nurse Team 4)

However, other teams find this ‘passive’ approach insufficient and emphasise the importance of ‘active’ managerial engagement. These teams prefer managers who are present during formal settings like morning briefings and during informal moments such as coffee breaks or lunches, fostering personal connections and gaining insights into the team's well‐being. One team expressed the need for personal attention beyond operational concerns.

It starts in the morning, not just asking about the number of beds and discharges, but simply saying good morning. How is the care burden? How are you doing? (Team 14)

The variability in expectations and satisfaction levels across teams suggests that a one‐size‐fits‐all approach to managerial support falls short. As one nurse noted, some colleagues feel that they do not see the manager ‘often enough’, although this is just a ‘personal perspective’. Such observations highlight the necessity for managers to tailor their approach to individual and team needs, as seen in our opening example.

Managers face the challenge of nurturing their teams while promoting certain levels of autonomy and independence. One manager cited research suggesting that healthcare professionals also desire care and support for themselves. However, this manager believes that excessive regulation and oversight can lead to ‘passivity’ within the team, aspiring instead to foster an ‘autonomous team environment’:

I would hope that the staff will gain more autonomy, allowing managers to take a step back, setting the boundaries, and enabling nurses to make their own choices within those parameters. (Manager Team 13)

For managers, finding the right approach to effective leadership can be challenging. One team manager noted from employee surveys that there was ‘low appreciation’ for leadership. After discussing this with the team, they suggested that increasing visibility on the floor might help. The manager said:

We already do a daily round at the start of the day, visit again around 11:30, and my door is always open. (Manager Team 14)

However, he wonders if the issue is truly about being more visible or if the team needs something else. The manager finds that the team often struggles to articulate what additional actions are required to feel more appreciated.

Additionally, managers observe that within a team, some nurses readily seek them out while others do not, making it challenging to find a ‘consistent approach’ to support. Another manager acknowledges having a distinct leadership style, openly communicating this to the team, which naturally fits better with some individuals than others:

There is a certain degree of wanting to be cared for that I do not want to indulge. I try to show my face every day, every shift, but some see it as insufficient. That's my style, and I always communicate that. It suits some better than others. (Manager Team 15)

This highlights the challenge of aligning individual leadership styles with the diverse needs of nursing teams, emphasising the importance of adaptability in managerial roles.

What stands out is that teams with alignment between managers and nurses, like the one in our opening example, have managed to establish a collaborative working relationship where both managerial and professional perspectives are acknowledged. Both parties recognise the challenges faced by the other and communicate these openly. The team understands the difficulties the manager encounters, and the manager is aware of the challenges the team faces. Together, they address these issues and discuss mutual expectations to improve the work environment from professional and managerial viewpoints.

## 6 Discussion

In this study, we explored how nursing teams organise their work environment by going beyond caregiving. Our findings highlight four themes – team features – that help nursing teams in creating a supportive nurse work environment: (1) embracing diversity, (2) stretching nursing roles, (3) raising voices, and (4) aligning nurses and managers. By developing these themes, nursing teams can better navigate the challenges of present‐day healthcare demands and work towards creating a professional nursing practise.

Our study highlights how diversity in nursing teams—shaped by differences in age and educational background—poses challenges and opportunities. Research indicates that generational groups assumptions can hinder understanding teammates' true capabilities, skills, information and connections (Gerhardt et al. 2021). However, *embracing diversity* can lead to positive effects, such as improved decision‐making, and negative effects, like subgroup formation (Homan et al. 2020). Nurses acting as organising professionals are expected to identify these dynamics and respond by implementing interventions, such as establishing shared team goals and valuing diverse perspectives (Homan et al. 2020). In doing so, they contribute to a work environment where diversity is seen as a strength, and people feel comfortable contributing. This is not easy, as individuals feel more comfortable with like‐minded peers (Fantahun et al. 2024). However, embracing multiple voices ultimately contributes to a more supportive work environment. To facilitate this, organisations may need to equip nurses as organising professionals with ‘diversity competencies’, such as understanding how diversity influences team dynamics (cognitive), developing skills to recognise emotions and reactions within the team (social), and developing flexibility to modify leadership strategies (behavioural) (Homan et al. 2020; Noordegraaf 2015).

Our research showed that some teams succeeded in *stretching nursing roles* by actively making their organisational contributions visible. This approach requires explicit acknowledgment of nurses' often‐invisible tasks and redefining and adopting professional roles (cf. Noordegraaf) better to reflect the evolving needs of healthcare organisations and society. However, within the teams in our study, we saw that it is often challenging for nurses to focus on roles beyond caregiving. Nurses frequently perform organisational tasks unknowingly, leaving this work invisible and unrecognised. Davina Allen (2014, 2023) refers to this ‘invisible work of nursing’ as tasks often performed between patient‐related activities or outside regular working hours that often go unnoticed. This lack of visibility means nurses rarely receive formal organisational support, such as allocated time, resources, coaching, or involvement in decision‐making processes. Similarly, informal support from colleagues—such as task‐sharing or patient coverage—is often limited (Kuijper et al. 2022). Thus, it is a challenge for nurses to make the stretching of nursing roles visible, redefine them, and adopt them.

In relation to *voice behaviour*, our research identified two key aspects: (1) the need for structured *platforms* that enable nurses to raise their voices and (2) the ability to *express* these voices, supported by enriched roles in relevant settings. Regarding the first key aspect, research indicates that hospitals increasingly embed nursing expertise into decision‐making processes through nursing councils and platforms. However, these initiatives are underutilised among nurses, which prevents them from connecting with others and the organisation (O'Grady and Clavelle 2021). Our study showed similar findings: many nursing teams still feel disconnected from such structures, struggling to see how these structures help them with their voice behaviour. This disconnection highlights the challenge of fostering stronger connections between nurses and the organisational structure. Additional studies emphasise the importance of approaches that are accessible, time‐efficient, and aligned with the nursing culture, while allowing for flexibility (Kremer et al. 2023; Klaassen 2022). As we consider the second key aspect, two concepts from the literature on voice behaviour may offer insights: *perceived efficacy—*the belief that speaking up will lead to meaningful change, and *perceived safety—*the confidence that voicing concerns will not result in retaliation or harm to relationships with colleagues or supervisors (van Dongen et al. 2024; O'Donovan et al. 2021). Our findings underscore the impact of perceived efficacy on nurses' willingness to speak up. For example, one team expressed frustration over the temporary closure of a department, noting that their input had not resulted in visible changes. This lack of impact diminished their confidence in speaking up. Similarly, some nurses highlighted concerns that the workplace environment did not feel safe enough to encourage open dialogue, further limiting voice behaviour.

The final theme, ‘aligning nurses and managers,’ refers to bringing together professional and managerial logic. Managers and nurses have a role in getting this done. Both need to share, substantiate, and combine their perspectives into joint action. In addition, managers have the responsibility to support and educate their nurses so the team is able to shape its work environment.

In our research, we saw managers struggle with their leadership actions, particularly in adapting them to the evolving needs and expectations of the team. This corresponds with earlier research that shows that managers play a crucial but often ambiguous role in supporting nurses in developing a more organising function within the profession (van Schothorst‐van Roekel et al. 2020). The ambiguity of this role refers to cooperating with nurses in aligning organisational and nursing aims and sometimes restricting nurses' active (organising) behaviour due to conflicting organisational concerns. To shape the nursing work environment, cultivating an interprofessional collaborative culture should be both the path and the goal (El‐Sayed et al. 2024).

The four themes identified in this study are interrelated; as team features, they can reinforce each other. When nurses adopt a stretched idea of nursing roles, they can more easily raise their voices when organising matters that extend bedside practices are at stake. Nurses gain insight into these organisational matters by using existing structures to make their voices heard. These matters become clearer when teams contain diversity, including diverse work experiences. Nursing teams that are able to cope with diversity in terms of age, experience, qualifications, and identities make differences more ‘productive’. Managers are important for guarding the conditions for such team practices, calling for nurses and managers to be ‘in tune’ with each other. Taken together, healthier team dynamics imply healthier work environments.

Our study contributes to the growing body of research on organising professionalism within nursing by examining how nursing teams can develop organising actions. Our findings build upon previous work, such as Felder et al.'s exploration of ‘partisan nurses’ (Felder et al. 2022), van Schothorst‐van Roekel et al.'s ethnographic account of nurse–manager relationships (van Schothorst‐van Roekel et al. 2020), and Postma et al.' study of neighbourhood nurses' articulation work (Postma et al. 2015). While these studies have illuminated various aspects of organising professionalism in nursing, including advocacy, role development, and community‐oriented approaches, our team‐focused perspective offers additional insights into fostering this role within the nursing profession.

### 6.1 Strengths and Limitations

One of the key strengths of this study is the comprehensive data collection. We were able to interview nearly all the teams in the hospital, providing a broad and representative view of the nurses' experiences and thoughts. The interviews were conducted in a safe and supportive setting, allowing participants to speak in‐depth about their experiences. To enhance the trustworthiness of the findings, we followed an iterative process in our thematic analysis, involving multiple researchers in coding and theme development. This approach helped ensure a more robust and consistent interpretation of the data. However, some limitations must be acknowledged. First, the participants in this study were already considered champions within their teams, which may have influenced their experience. These individuals likely had a more favourable or proactive view of their organisation compared with their colleagues. Additionally, the presence of team managers alongside nurses in the focus groups may have affected the dynamics and openness of discussions. This combination of participants could have led to potential power imbalances or reluctance to express certain views. To mitigate these biases, we explicitly asked participants to describe what happens in the nursing team and encouraged open dialogue regardless of role. Two interviews were conducted without a manager present, as the managers were unable to attend at the last minute. We cannot ascertain whether this absence had any impact on the findings. Second, the study mainly relied on interviews, meaning that the findings are based on self‐reported data; we did not use triangulation with other data sources, such as document analysis or formal observations. Although this could affect the internal validity, the fact that we held group interviews corrected this. The fact that we have immersed ourselves in the hospital context implies that we were well aware of ‘real’ dynamics and were able to ‘get beyond’ potentially biased answers. Finally, since this study was conducted in a single hospital, the generalisability of our findings is limited. Therefore, our findings should be generalised with caution. Future studies should involve different hospitals or even different countries to gain a broader understanding of how nursing teams reshape their work environments.

## 7 Conclusion

In this study, we explored how nursing teams organise their work environment by going beyond ‘bedside work’ and caregiving. We found that shaping a supportive work environment is a process that requires intentional effort and active collaboration within the team and with managers. Four team features contributing to such efforts were identified: embracing diversity, stretching nursing roles, raising voices, and aligning nurses and managers. These team features can reinforce each other. Together, they contribute to a healthier work environments, and ultimately, better care. Paradoxically, improving caregiving begins by going beyond caregiving itself.

### 7.1 Implications for Policy and Practice

The four team features can help nursing teams reflect on their organising practices and identify areas for development, such as team diversity, voicing, role stretching, and alignment. Nurses can use these insights to support team development. For management, it is important to recognise, appreciate, and enlarge nurse roles that extend beyond direct caregiving to improve caregiving. This implies that management must go beyond directly managing work environments to improve work environments.

### 7.2 Recommendations for Further Research

Future research could further define and operationalise the concept of organising professionalism by identifying the specific capabilities and practices that enable nurses to contribute to shaping their work environments. This includes exploring how these capabilities can be supported and developed across different healthcare settings. It is also important to examine the tensions, barriers, and enabling conditions that arise when nursing teams take more active roles in organising their work. Practice‐oriented research methods such as action research are particularly suitable, as they allow for interactive and adaptive collaboration between researchers, nurses, and managers in testing and strengthening organising professionalism in everyday practice.

## Conflicts of Interest

The authors declare no conflicts of interest.

## Data Availability

The data that support the findings of this study are available on request from the corresponding author. The data are not publicly available due to privacy or ethical restrictions.

## Appendix 1

*Topic list*

**Table:**

Section	Content
Start	Begin with a brief introduction and review the *Excellent Care* program Discuss the current program status and introduce the new focus: work environment on team level
Reflection	Rank the eight characteristics of team satisfaction from ‘most satisfied’ to ‘least satisfied’:— Reflect on each characteristic during the ranking process, explaining why each card was placed in its position. working with clinically competent peers collaborative nurse–physician relationships clinical autonomy nurse manager support control over nursing practice perceived adequacy of staffing support for education culture in which attention for the patient is paramount Assign a score to the highest and lowest‐ranked characteristics Define a threshold that distinguishes between sufficient and insufficient satisfaction levels
Final question	Imagine revisiting this reflection 6 months from now. How would you rank the characteristics at that time, and what actions would you take to reach that desired outcome?
